# [CrF(O_2_C^*t*^Bu)_2_]_9_: Synthesis and Characterization of a Regular Homometallic Ring with an Odd Number of Metal Centers and Electrons

**DOI:** 10.1002/anie.201601734

**Published:** 2016-06-13

**Authors:** Robert J. Woolfson, Grigore A. Timco, Alessandro Chiesa, Inigo J. Vitorica‐Yrezabal, Floriana Tuna, Tatiana Guidi, Eva Pavarini, Paolo Santini, Stefano Carretta, Richard E. P. Winpenny

**Affiliations:** ^1^ School of Chemistry and Photon Science InstituteUniversity of ManchesterOxford RoadManchesterM20 4NZUK; ^2^ Dipartimento di Fisica e Scienze della TerraUniversità di ParmaParco Area della Scienze 7/AI-43124ParmaItaly; ^3^ ISIS FacilityRutherford Appleton LaboratoryDidcotOX11 0QXUK; ^4^ Institute for Advanced SimulationForschungzentrum Jülich52425JülichGermany; ^5^ JARA High Performance ComputingRWTH Aachen University52062AachenGermany

**Keywords:** inelastic neutron scattering, magnetic properties, molecular magnetism, spin frustration, supramolecular chemistry

## Abstract

The first regular homometallic ring containing an odd number of metal centers is reported. The ring was synthesized by means of amine‐templated self‐assembly. Extensive physical characterization studies, including magnetic measurements, powder inelastic neutron scattering (INS), and DFT calculations, show that the molecule has a near perfect match to the expected behavior for a frustrated system with the lowest energy pair of *S*=1/2 spin states separated by only 0.1 meV (0.8 cm^−1^).

The literature on polymetallic compounds with cyclic metal cores has certain unusual gaps. Since the publication in 1990 of both {Fe_10_}^1^ by Lippard and Taft and {Cr_8_}^2^ by Timco and co‐workers, a wide range of sizes have been reported for homometallic rings, including {Fe_18_}/{Ga_18_}^3^ rings (currently the largest reported regular wheels, that is, where every edge and every vertex is chemically equivalent) to the more complex {Mn_32_} double decker structure.^4^ “Magic number” rings of {Mn_84_}^5^ and {Pd_84_}^6^ have also been reported. The largest cyclic metal structures remain the extraordinary molybdate wheels from Müller and co‐workers.^7^ However, rings with odd numbers of metal centers (termed herein odd‐numbered rings), particularly those larger than triangles, are conspicuous by their near absence.

There are a few reported heterometallic nine‐metal rings, including {Cr_7_V_2_} and a {Cr_8_M} family.^8^ However, regular homometallic rings containing odd numbers of metal centers are restricted to metal triangles, a small number of pentagons,^9^, ^10^ and a single heptametallic {V_7_} ring made by Oshio and co‐workers with the vanadium sites bridged by a cyclodextrin ligand.^11^

Large regular odd‐numbered rings would show interesting physics associated with spin frustration,^12^, ^13^ which occurs when the classical magnetic energy cannot be simultaneously minimized for all individual two‐spin interaction terms. Theoretical studies to classify the degree of frustration in such systems have been recently reported.^14^ In 2002 the structures of two toroidal thiolate‐bridged nickel structures, {Ni_9_} and {Ni_11_}, were published by Dahl and co‐workers, both of which showed considerable geometric irregularity; however, no characterization beyond crystallography was reported.^15^ In 2004, Mezei et al. reported a nonametallic copper ring {Cu_9_} that was not isolated but co‐crystallized with a range of other copper rings.^16^

We previously reported two nonametallic rings {Cr_9_F_10_} and {Cr_9_F_11_} (see below), each of which contained one edge with a defect breaking the regularity of the ring.^17^ Herein, we report the synthesis of the first regular nine‐metal homometallic ring, [CrF(O_2_C^*t*^Bu)_2_]_9_ **1**, along with characterization of the ring by magnetometry and inelastic neutron scattering (INS) to explore the extent of spin frustration.

The synthesis of **1** is based on a procedure we previously developed for synthesizing hexametallic and heptametallic chromium horseshoes^18^—addition of a templating amine to a reaction that would conventionally give [CrF(O_2_C^*t*^Bu)_2_]_8_ **2** (see Figure S1 in the Supporting Information). Using diisopropylamine as the template along with the addition of trifluoromethanesulfonic acid leads to a mixture of octa‐ and nonametallic rings which can be purified by column chromatography, resulting in the separation of **1** and **2**, along with the previously published^17^ [NH_2_^*i*^Pr_2_][Cr_9_F_10_(O_2_C^*t*^Bu)_18_] (**3**; {Cr_9_F_10_}) and [NH_2_^*i*^Pr_2_][Cr_9_F_11_(O_2_C^*t*^Bu)_17_] (**4**; {Cr_9_F_11_}; see Figure S6). The templating ammonium cation is not present in **1** but addition of diisopropylamine is essential for the compound to form.

Crystals of **1** can be grown from a range of solvents (toluene, acetone, *n*‐propanol) resulting in two distinct types of crystals. Crystals with hexagonal space group *P*6_3_/*m* show significant disorder in the pivalate carbons and only the Cr, F, and O positions can be refined with confidence. However, crystals with the orthorhombic space group *Pcba* can be grown from *n*‐propanol and the full crystal structure determined (Figure 1). A list of average Cr−Cr and Cr−F bond lengths as well as average bridging Cr‐F‐Cr angles for **1**–**4** is given in Table 1.

**Figure 1:**
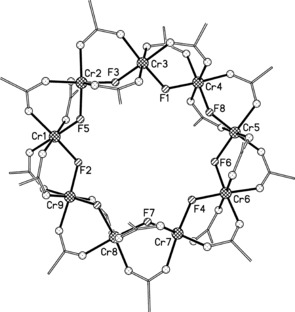
Crystal structure of **1**, crystals grown from *n*‐propanol. Cr (cross‐hatched circles), F (striped circles), O (dotted circles), C (lines). Methyl groups of pivalates and solvent molecules are omitted for clarity.

**Table 1:** Selected average bond lengths [Å] and angles [°] for compounds **1**–**4**.

	Compound	Cr−Cr bond^[a]^	Cr−F bond^[a]^	Cr‐F‐Cr angle^[a]^	
	**1**	3.376±0.016 (0.052)	1.918±0.013 (0.044)	123.300±0.748 (2.100)	
	**2**	3.383±0.018 (0.051)	1.916±0.006 (0.023)	124.000±0.735 (0.051)	
	**3**	3.390±0.049 (0.158)	1.929±0.012 (0.041)	123.067±3.304 (11.000)	
	**4**	3.391±0.047 (0.164)	1.927±0.016 (0.061)	123.278±2.952 (9.700)	

[a] The largest difference in value for each parameter is given in parentheses.

Structurally, **1** is more closely related to the regular octagon **2** rather than nine‐metal rings **3** and **4** that each have a unique edge breaking the regularity of the metal polygon. The chromium atoms in **1** do not lie in a plane but rather take a bowl‐shaped conformation. Each chromium site has a slightly distorted octahedral coordination environment. The Cr−Cr distances and Cr‐F‐Cr angles are very regular, as in **2** (see Table 1). Unlike **3** and **4**, **1** is neutral and therefore solvent, rather than the templating amine, occupies the central cavity.

Given the regular structure of **1**, we have studied samples of **1** recrystallized from toluene to assess the degree to which it shows spin frustration. According to the strict definition given by Kahn,^19^ frustration would manifest itself as the presence of two degenerate *S*=1/2 ground multiplets. Magnetic measurements, specifically variable‐temperature susceptibility (*χ_m_*(*T*); Figure S7) and variable‐field magnetization (*M*(*H*); Figure S8), show the expected behavior for an antiferromagnetic exchange between the Cr^III^ centers in **1**, and can be fitted to a Hamiltonian containing a single antiferromagnetic exchange parameter *J*=1.35(3) meV (1 meV=8.066 cm^−1^). These thermodynamic measurements confirm the presence of an *S*=1/2 ground state and support the presence of spin frustration, but they are comparatively insensitive compared with spectroscopic techniques. The size of the exchange coupling is somewhat smaller than that found in **2** which is 1.46 meV.^20^

Inelastic neutron scattering (INS) probes the spin dynamics of the system and can directly detect any splitting of the pair of potentially degenerate ground‐state doublets. INS data were collected using the low energy transfer (LET) spectrometer on a powder sample of **1** recrystallized from toluene (Figure 2, top), using 7.48 meV incident neutron energy (*E_i_*) measured at 1.5, 7, and 15 K. The dominant peak at low temperature corresponds to the transition between the ground *S*=1/2 states and the lowest energy *S*=3/2 multiplet, labelled transition I. At higher resolution (*E_i_*=2.5 meV), a splitting of this peak of about 0.1 meV is detected (Figure 3). Higher‐energy peaks are detected at all temperatures, corresponding to transitions from the *S*=1/2 ground state to higher‐energy excited states with *S*=3/2 (transitions II and V in Figure 2, bottom) or *S*=1/2 (transitions III and IV, respectively). At 15 K, hot transitions can be detected due to additional excitations from the lowest energy *S*=3/2 state (transitions VI and VII). The seemingly temperature‐independent broad peak at 3 meV is due to an overlap of two peaks corresponding to one cold (III) and one hot transition (VIII). All these results are well reproduced by a Hamiltonian with *C*_9_ symmetry, thus demonstrating that the spin dynamics of **1** matches that expected of a perfectly frustrated antiferromagnetic ring (a spectrum calculated with a *C*_9_ Hamiltonian is reported in Figure S9).

**Figure 2:**
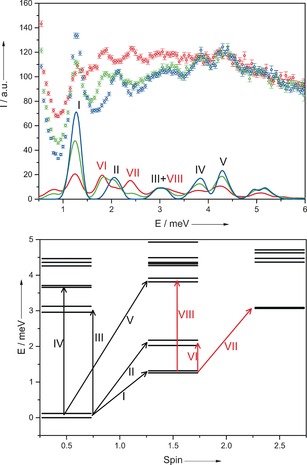
Top: Powder INS data for **1** recorded on an LET spectrometer using 7.48 meV incident neutron energy. Experimental (open circles) and simulated (solid lines) spectra shown for 1.5 K (blue), 7 K (green), and 15 K (red) with peaks labelled corresponding to transitions in the diagram below. Bottom: Isotropic exchange diagram for **1** derived using calculated *J*=1.32 meV (10.6 cm^−1^) and *J*′/*J*=1.2.

**Figure 3:**
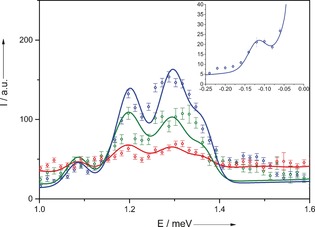
INS spectra recorded using *E_i_*=2.5 meV incident energy neutrons. Experimental (open circles) and simulated (solid lines) spectra at 1.5 K (blue), 7 K (green), and 15 K (red) are shown. A background has been added to the simulated curves. Inset: Experimental and simulated data for the *E_i_*=1.24 meV (10.0 cm^−1^) spectrum showing an intra‐multiplet *S*=1/2 transition.

To investigate the degree of frustration in **1** quantitatively, we performed high‐resolution (*E_i_*=1.24 meV and 22 μeV full‐width half‐maximum, FWHM, at elastic line) INS measurements which revealed a small (0.1 meV) splitting between the two *S*=1/2 ground states (Figure 3, inset), indicating a slight lifting of the degeneracy in **1** and thus a small removal of frustration from that of a perfectly frustrated ring.

All the INS data can be well reproduced using the minimal Hamiltonian in Equation [1], where for simplicity we model the splitting of the lowest‐energy doublets by breaking the *C*_9_ symmetry of the enneagon on one edge with the introduction of a second exchange constant, *J*′.

(1) $$\;H=\sum_{i=1}^{8}J{𝐬}_{i}\cdot {𝐬}_{i+1}+J^{'}{𝐬}_{9}\cdot \;{𝐬}_{1}+d\sum_{i}s_{z_{i}}^{2}+\sum_{i>j=1}^{9}{𝐬}_{i}\cdot D_{ij}\cdot \;{𝐬}_{j}$$

In this Hamiltonian, ***s**_i_* are spin operators for site *i* and *d* is the axial single‐ion zero‐field‐splitting parameter. The last term describes intramolecular magnetic dipole–dipole interactions, with couplings *D_ij_* calculated in the point‐dipole approximation (no additional free parameters). Fitting the INS data resulted in parameters of *J*=1.32 meV (10.6 cm^−1^), *J*′=1.2⋅*J*=1.58 meV and *d*=−0.022 meV. It is worth noting that the average value of the exchange constants is the same found from magnetometry. This model reproduces the observed splitting of the peak corresponding to transition I (*S*=1/2→*S*=3/2; Figure 3), which is due to the combined action of the zero‐field splitting and of the slight lifting of the ideal ring symmetry. Figure 2 (bottom) shows the energy‐level diagram for **1** calculated with the isotropic part of Equation [1]. The measured dependence of the neutron intensity on the momentum transfer *Q* for the main peak I (see Figure S10) is in good agreement with calculations, confirming that the structure of the states involved in the transition is well reproduced by our model.

To validate our findings, we have performed density functional theory (DFT) calculations employing the NWChem software,^21^ using the broken symmetry approach to compute the isotropic exchange couplings (see the Supporting Information for details). Calculations have been done on the Jülich supercomputer Jureca. An average *J* value of 1.40 meV is found from these calcuations, which is in very good agreement with that obtained by fitting magnetometry and INS data (1.35 meV). The distribution of the calculated couplings is much more uniform than those of the previously reported Cr9 wheels,^17^ with a small standard deviation of 0.17 meV.

The internal spin structure of the ground state of **1** has been investigated through analysis of the scalar spin chirality $\hat{C}$ [Eq. (2)], which is a measure of the planarity of the spin configuration.

(2) $$\hat{C}=\sum_{i=1}^{9}{𝐬}_{i}\cdot {𝐬}_{i+1}\times {𝐬}_{i+2}$$

The results show that the quantum ground state of **1** is an equal superposition of chirality eigenstates with opposite eigenvalues (see Figures S11 and S12). Thus, in contrast to the classical spin configurations (in which all of the spins lie in the same plane), Cr_9_ fluctuates between nonplanar states with opposite chirality keeping  *C*>=0, consistent with the behavior expected for an ideally frustrated antiferromagnetic ring.

Compound **1** is the first compound reported containing a regular homometallic ring containing nine metal centers. The INS data for the compound reveal nearly frustrated spin dynamics, with a slight lifting of the degeneracy of the lowest‐energy states resulting in a splitting of the two *S*=1/2 multiplets of only 0.1 meV. Compared to systems such as triangles where highly rigid ligand systems are required to ensure spin frustration,^22^ obtaining a near perfect frustrated ground state from such a sterically undemanding set of starting materials is remarkable. Given the strong evidence for spin frustration in **1**, further studies of the spin properties, such as spin density or correlation dynamics, will provide fascinating information on the physics of such large frustrated spin systems.

## Supporting information

As a service to our authors and readers, this journal provides supporting information supplied by the authors. Such materials are peer reviewed and may be re‐organized for online delivery, but are not copy‐edited or typeset. Technical support issues arising from supporting information (other than missing files) should be addressed to the authors.

Supplementary
