# Supplementary material for: [CrF(O2CtBu)2]9: Synthesis and Characterization of a Regular Homometallic Ring with an Odd Number of Metal Centers and Electrons
Source: Angew Chem Int Ed Engl. 2016 Jun 13;55(31):8856–9. doi: 10.1002/anie.201601734 (PMC5089673; doi:10.1002/anie.201601734)
Supplement: Supplementary file 1 — Supplementary [file ANIE-55-8856-s001.pdf]

Angewandte Chemie
Eine Zeitschrift der Gesellschaft Deutscher Chemiker

Supporting Information

$[CrF(O_2C^tBu)_2]_9$: Synthesis and Characterization of a Regular Homometallic Ring with an Odd Number of Metal Centers and Electrons

*Robert J. Woolfson, Grigore A. Timco, Alessandro Chiesa, Inigo J. Vitorica-Yrezabal, Floriana Tuna, Tatiana Guidi, Eva Pavarini, Paolo Santini, Stefano Carretta, and Richard E. P. Winpenny**

anie_201601734_sm_miscellaneous_information.pdf

SUPPLEMENTARY INFORMATION

1. Experimental Section

All reagents and solvents were purchased from commercial sources and used as received. Reactions were performed in Erlenmeyer Teflon flasks. Column chromatography was performed using 45-60 μm Silica as a gel with wet loading in toluene.

Pivalic acid (40 g, 396 mmol), chromium (III) trifluoride hexahydrate (5.0 g, 27.61 mmol) di-*iso*propylamine (0.58 g, 0.81 mL, 5.73 mmol) and trifluoromethanesulfonic acid (0.42 g, 0.25 ml, 3.68 mmol) were stirred together at 150 °C for 24 h. The flask was allowed to cool for 15 m, MeCN (100 mL) added and the solution stirred for 2 h resulting in a solid green precipitate. This was collected by filtration, washed with MeCN and then extracted with acetone (500 mL) while stirring overnight. The resulting solution was filtered and then evaporated to dryness by rotary evaporation. The green powder contained a mix of **1, 2, 3** and **4** that was separated by column chromatography. With toluene as the eluent, **2** eluted first followed more slowly by **1**. Increasing the polarity to 40:1 toluene:ethyl acetate resulted in elution of **3** and 10:1 toluene:ethyl acetate eluted **4**. See reference 17 in main text for physical characterisation of **3** and **4**. CCDC entries 164814, 893834 and 893835 contain the supplementary crystal data for **2**, **3** and **4**.

Evaporation of the fraction containing **1** gave a green powder with a yield of 0.21 g (2.7 %). Positive ion electrospray mass spectrometry (FTMS+) using THF as carrier solvent gave m/z values of 2460 (M + H); 2477 (M + H + H_2O); 2492 (M + H + MeOH); 2513 (M + Na + MeOH); 2520 (M + H + iso-propanol); 2533 (M + H + THF); 2556 (M + pivalate) (Figure S1). Water, methanol, iso-propanol and sodium are common ions observed in our mass spectrometer and THF was the carrier solvent. Elemental analysis (calculated): Cr 18.73 (19.03), C 43.76 (43.96), H 6.60 (6.64) N 0 (0). ATIR (cm^{-1}): 2966 (C-H stretch); 1552; 1484; 1427; 1363; 1229; 768; 613 (Figures S2 and S3).

Single crystals of **1** suitable for X-ray diffraction were grown from either – (1) slow evaporation of a saturated *n*-propanol solution for orthorhombic crystals, (2) slow evaporation of acetone for monoclinic crystals or (3) slow evaporation of toluene for hexagonal crystals.

INS data were collected using the LET[1] time-of-flight spectrometer at ISIS, Rutherford Appleton Laboratory, United Kingdom, on a 0.4 g of polycrystalline non-deuterated sample crystallised from toluene and powdered. The sample was wrapped in aluminium foil and then loaded into a standard orange cryostat for measurements. Measurements were performed at 1.5, 7 and 15 K with 7.48, 2.5 and 1.24 meV incident neutron energies using the repetition rate multiplication mode.

Magnetic characterisation of polycrystalline **1** was performed in the temperature range 1.8 – 300 K using a Quantum Design MPMS XL SQUID magnetometer equipped with a 7 T magnet. Diamagnetic correction for the sample was calculated using Pascal's constants and measured for the sample holder.

1.1. Mass Spectrometry

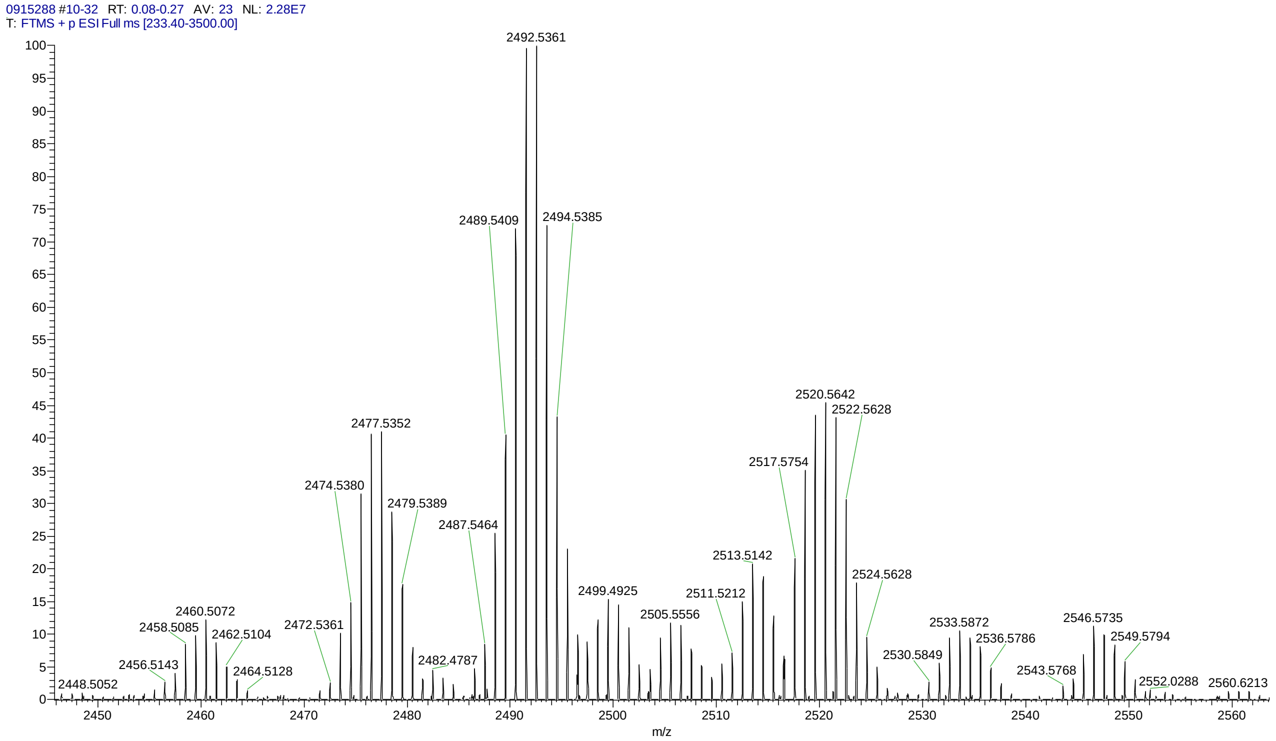

Figure S1: FTMS+ spectrum for **1**

1.2. Infra-red Spectra

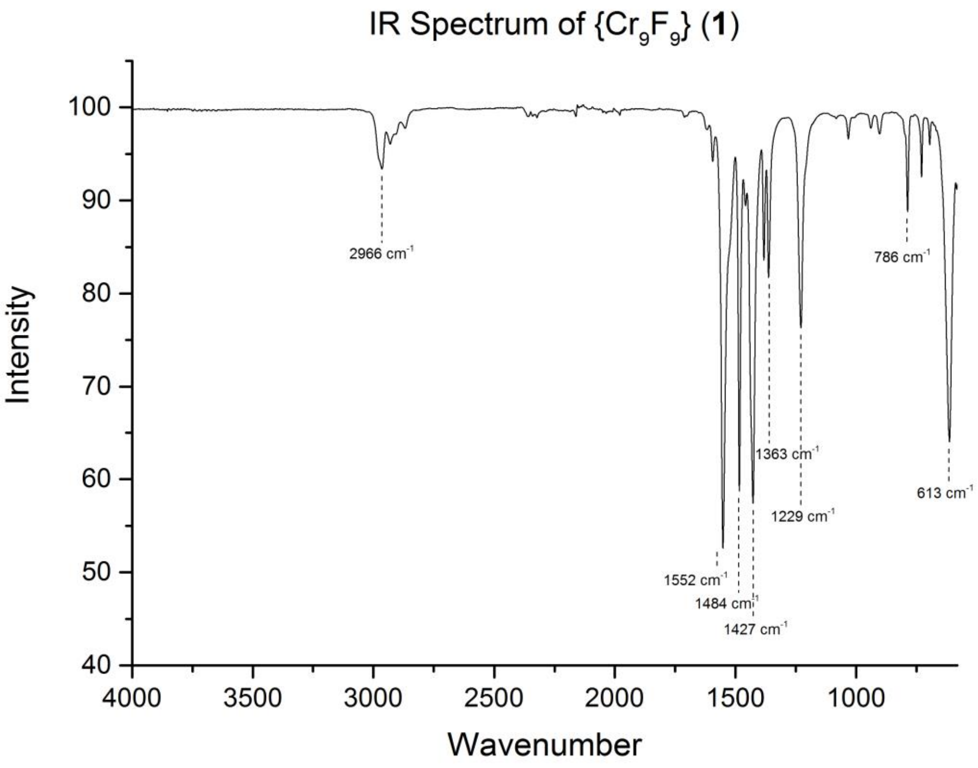

Figure S2: Infra-red spectrum of **2**

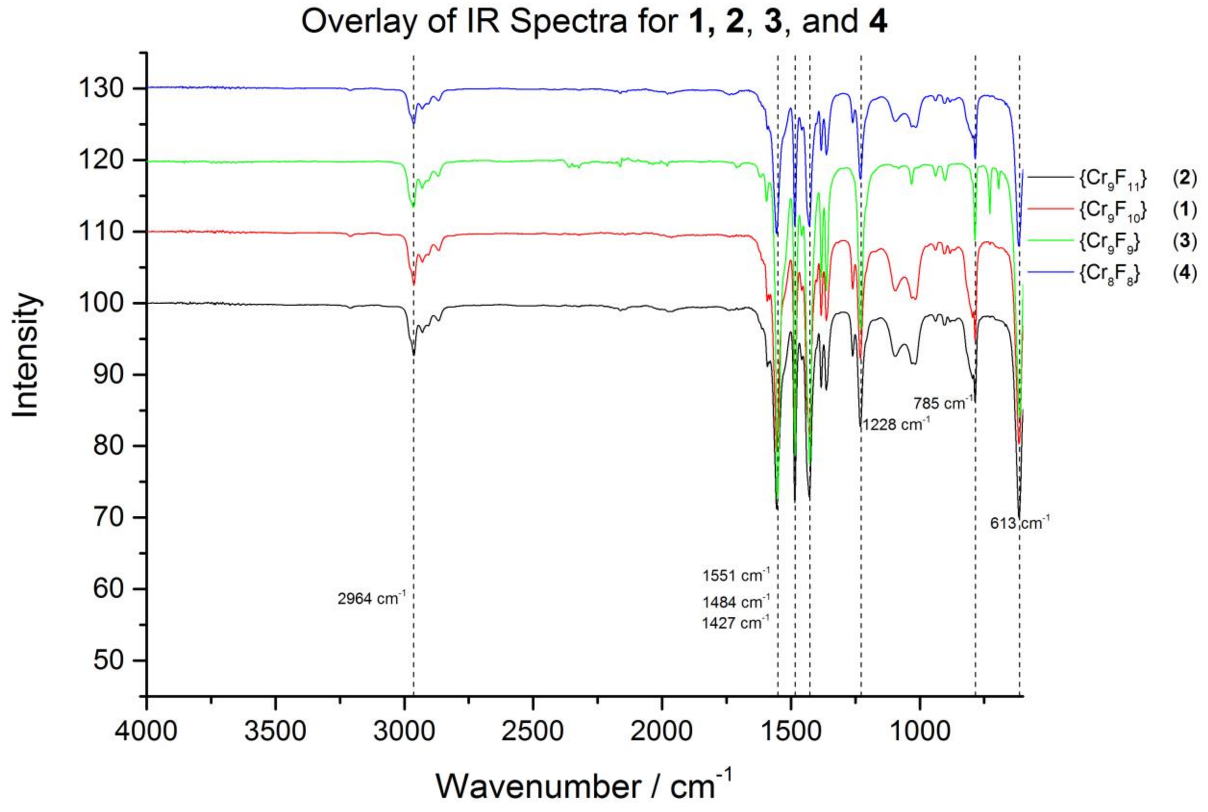

Figure S3: Overlay of the infra-red spectra for **1**, **2**, **3** and **4** showing no significant differences in the observed spectra of the four compounds.

2. Crystallography

Data Collection. X-ray data for compound **1/n-propanol** was collected at a temperature of 150 K using a using Mo-K_{α} radiation on a microfocus Agilent Supernova diffractometer, equipped with an Oxford Cryosystems Cobra nitrogen flow gas system. Data were measured using CrysAlisPro suite of programs. X-ray data for compound **1/acetone** was collected at a temperature of 100 K using Cu-K_{α} radiation on a microfocus Bruker X8 prospector diffractometer, equipped with an Oxford Cryosystems Cobra nitrogen flow gas system. Data were measured using Apex2 suite of programs. X-ray data for compound **1/toluene** was collected at beamline I19 in Diamond Light Source at a temperature of 100 K using synchrotron radiation (λ = 0.6889),[2] equipped with an Oxford Cryosystems Cobra nitrogen flow gas system. Data were measured using CrystalClear-SM Expert 2.0 r5 suite of programs.

Crystal structure determinations and refinements. X-ray data were processed and reduced using CrysAlisPro suite of programs. Absorption correction was performed using empirical methods based upon symmetry-equivalent reflections combined with measurements at different azimuthal angles.[3] The crystal structure was solved and refined against all F^2 values using the SHELX and Olex2 suite of programs.[4] Atoms corresponding to compound **1/n-propanol** and **1/acetone** were refined anisotropically. Acetone solvent molecules and some of the pivalates in compound **1/acetone** were refined isotropically in order to keep the data/parameter ration as high as possible. Hydrogen atoms were placed in calculated positions refined using idealized geometries (riding model) and assigned fixed isotropic displacement parameters. Pivalates ligands were highly disorder. The C-C distances of the pivalate ligands were restrained to be the same using DFIX and SADI commands. The atomic displacement parameters (adp) of the ligands have been restrained using RIGU and SIMU commands.

Compound **1/acetone** presents a large amount scattered electron density, the SQUEEZE protocol inside PLATON suites was used to account the electron density.[5] The results of the SQUEEZE protocol indicated that the remaining electron density correspond to two disordered molecules of acetone.

A large number of A and B alerts were found in compound **1/acetone** due to poor resolution (1.1 Å) data. Poor resolution data affects the shapes of the electron density maps obtained, and consequently to the accuracy of the model produced.

CCDC entry 1421216 contains the supplementary crystal data for **1/n-propanol** and entry 1470536 contains the crystal data for **1/acetone**.

Single crystal X-ray diffraction data for **1/toluene** was collected in both “in-house” diffractometers and with synchrotron radiation. Crystals only diffracted until 1.4 Å of resolution at I19 beamline in Diamond Light Source. As we can see in the Figure S4, smeared diffraction spots formed the diffraction pattern obtained. Decrease of the scan width or data collection at different temperatures did not improve the quality of the diffraction patterns. Due to the extremely complex disorder of the Cr9 rings in the crystal packing, the structure remains unsolved and it is impossible to discuss any structural characteristics of the Cr9 rings. However as the ring can be recrystallised intact from two other solvents we are confident they are intact in crystals grown from toluene.

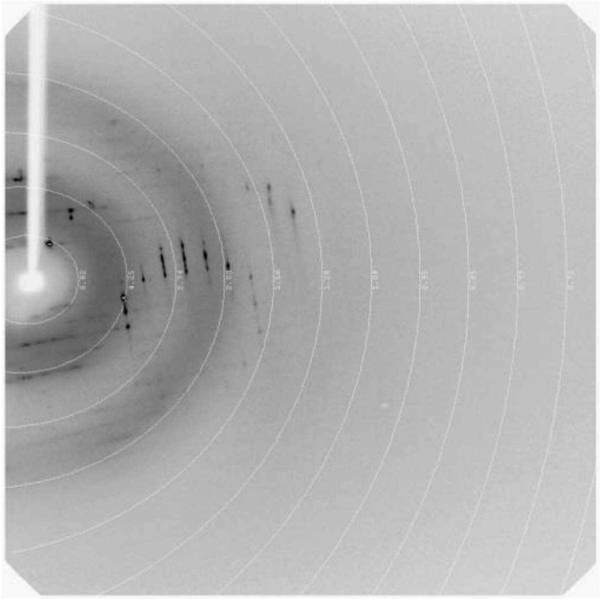

Figure S4. Diffraction pattern of **1/toluene** obtained at beamline I19 in Diamond Light Source.

Nevertheless, the crystal packing of both **1/acetone** and **1/toluene** present some similarities. As we can see in Figure S5, the crystal packing of **1/toluene** in the (001) plane S5a) and (010) plane S5c) are similar to the crystal packing of **1/acetone** in the (001) plane S5b) and (101) plane S5d). We can also appreciate how the molecules of compound **1/acetone** are tilted with respect to the (001) and (101) planes in the crystal structure. The angles formed by the molecules with respect to these planes are a consequence of the internal symmetry dictated by the space group. **1/toluene** molecules shown in the pictures are perfectly parallel to the (001) and (010) planes. The ellipsoids generated in **1/toluene** are extraordinary large, suggesting that the molecules of **1** are randomly disordered above and below the (001) and (010) planes. Therefore, it can be speculated that the diffused diffraction spots obtained in the diffraction pattern are consequence of the severe disordered found in **1/toluene.**

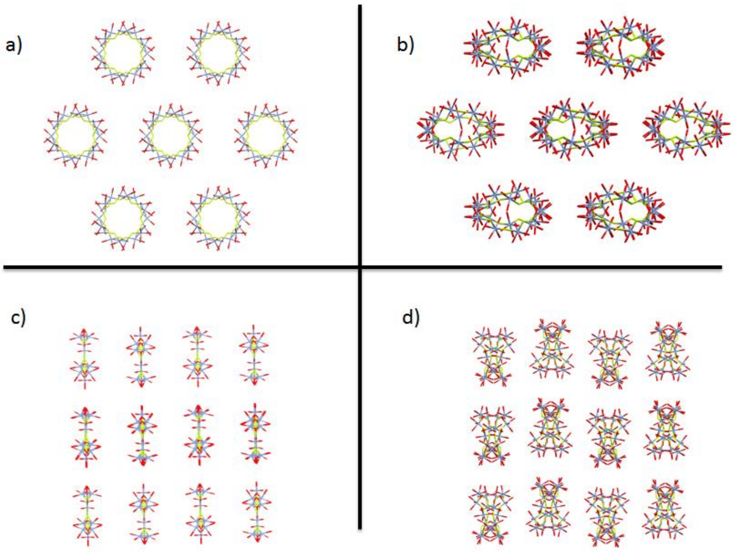

Figure S5. Crystal packing view of a) **1/toluene** plane (001); b) **1/acetone** plane (001); c) **1/toluene** plane (010); and d) **1/acetone** plane (101)

Table S1. Crystallographic information for **1/n-propanol, 1/acetone** and **1/toluene**

	**1/n-propanol**	**1/acetone**	**1/toluene**
Crystal colour	green	green	green
Crystal size (mm)	0.35 × 0.2 × 0.07	0.3 × 0.15 × 0.08	0.15 × 0.15 × 0.15
Crystal system	Orthorombic	Monoclinic	Hexagonal
Space group, *Z*	Pbca, 8	$P2_1/n$, *4*	$P6_3/m$, *6*
a (Å)	30.5495(5)	19.2354(8)	19.4172(6)
b (Å)	25.4613(7)	24.077(2)	19.4172(6)
c (Å)	34.4733(9)	32.214(2)	26.310(3)
β (°)	90	90.937(4)	90
V (Å^3)	26814(1)	14918(2)	7937(1)
Density (Mg.m^{-3})	1.248	1.198	
Wavelength (Å)	0.71073	1.54718	0.6889
Temperature (K)	150	100	100
μ(Mo-Kα) (mm^{-1})	0.778	5.836	
2Θ range (°)	6.302 to 52.768	6.458 to 79.94	
Reflns collected	118555	26536	70044
Independent reflns (R_{int})	27371 (0.098)	9002 (0.1163)	5520 (0.2138)
L.S. parameters, *p*	1727	1120	
No. of restraints, *r*	1688	2604	
$R1$ $(F)^a$ $I > 2.0\sigma(I)$	0.0949	0.1390	
$wR2(F^2)$,^{a} all data	0.3048	0.3900	
$S(F^2)$,^{a} all data	1.056	1.428	

^{a} $R1(F) = \Sigma(|F_o| - |F_c|)/\Sigma|F_o|$; [b] $wR^2(F^2) = [\Sigma w(F_o^2 - F_c^2)^2/\Sigma wF_o^4]^{½}$; [c] $S(F^2) = [\Sigma w(F_o^2 - F_c^2)^2/(n + r - p)]^{½}$

2.1. **Structures of compounds 2,3 and 4**

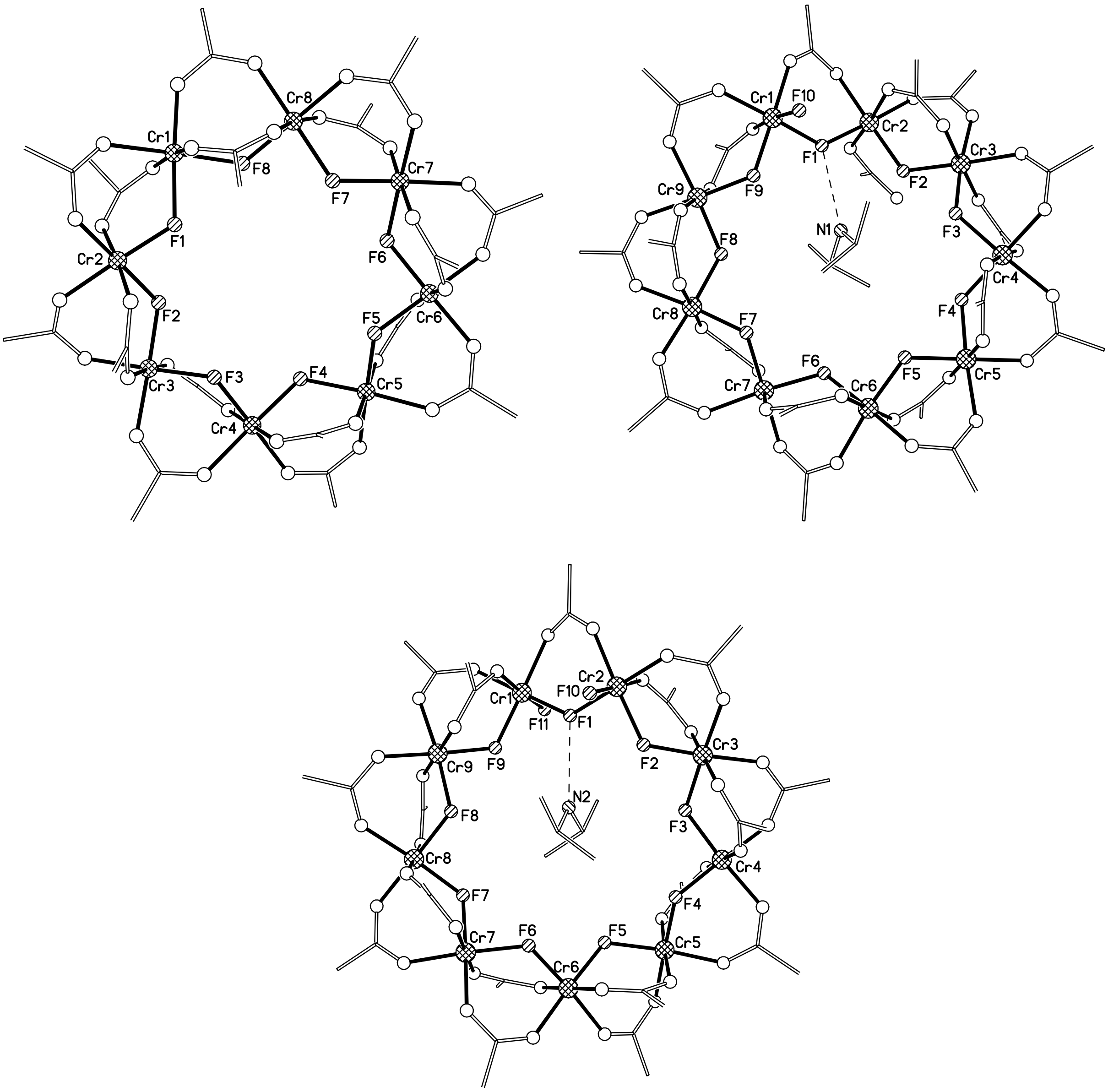

Figure S6: The structures of compounds **2** (top left), **3** (top right) and **4** (bottom). The inequivalent edge in **3** and **4** is at the top of each molecule and contains F1. Cr (cross-hatched circles), F (striped), O (dotted circles), C (lines). Methyl groups of pivalates and solvent molecules omitted for clarity.

3. Magnetisation

Magnetic data were simulated using the isotropic Hamiltonian below with the parameters given in the main text (J = 1.32 meV, J' = 1.58 meV).

$$H = \sum_{i=1}^{8} J\, \boldsymbol{s}_i \cdot \boldsymbol{s}_{i+1} + J'\, \boldsymbol{s}_9 \cdot \boldsymbol{s}_1$$

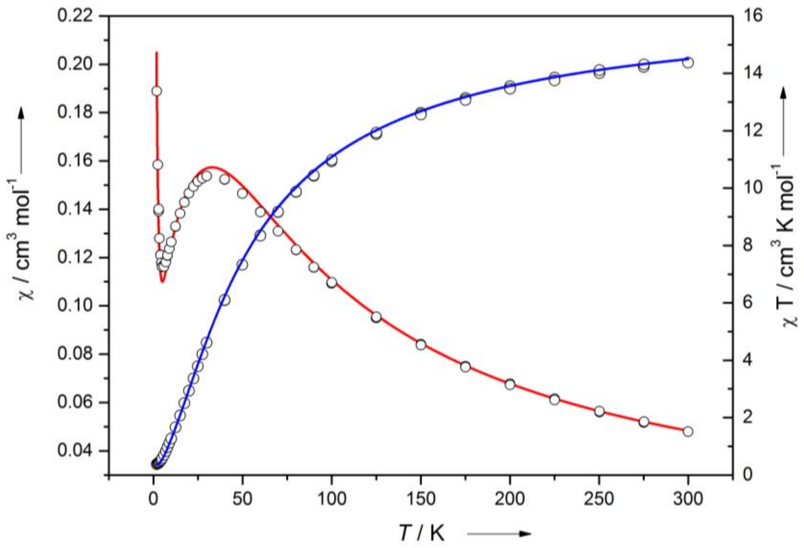

Figure S7: Magnetic susceptibility (χ) versus temperature (T) and χT versus T for compound 1 with data (open circles) and calculation (solid lines).

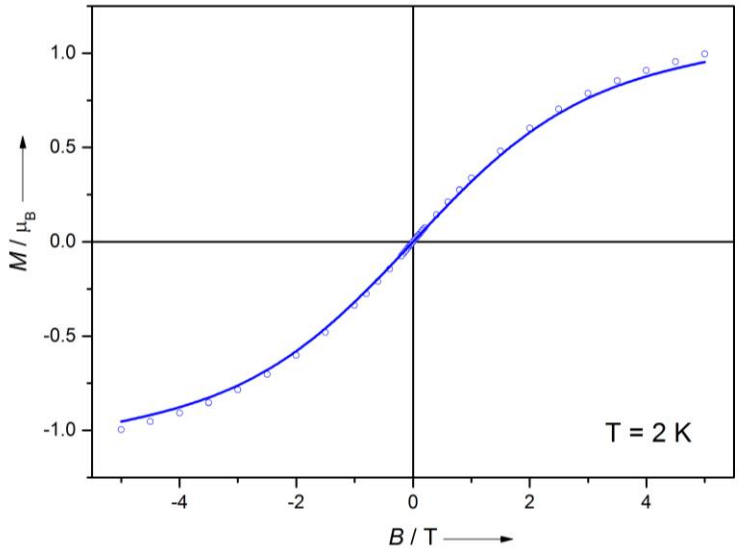

Figure S8: Magnetization (M) versus field (B) for compound **1** with simulation (solid line).

4. INELASTIC NEUTRON SCATTERING

Figure S9 below reports the comparison between the fit of the E_i =2.5 meV data with an ideally frustrated model (uniform *J* couplings, left panel) and the one reported in Eq. 1 of the main text (J'/J=1.2, right). Although the detailed splitting of the peak is better reproduced by using spin Hamiltonian (1), the main features are captured by the frustrated model Hamiltonian. In addition, higher-energy peaks are well reproduced by both models. This shows that the spin dynamics of the system is essentially the same of a frustrated ring.

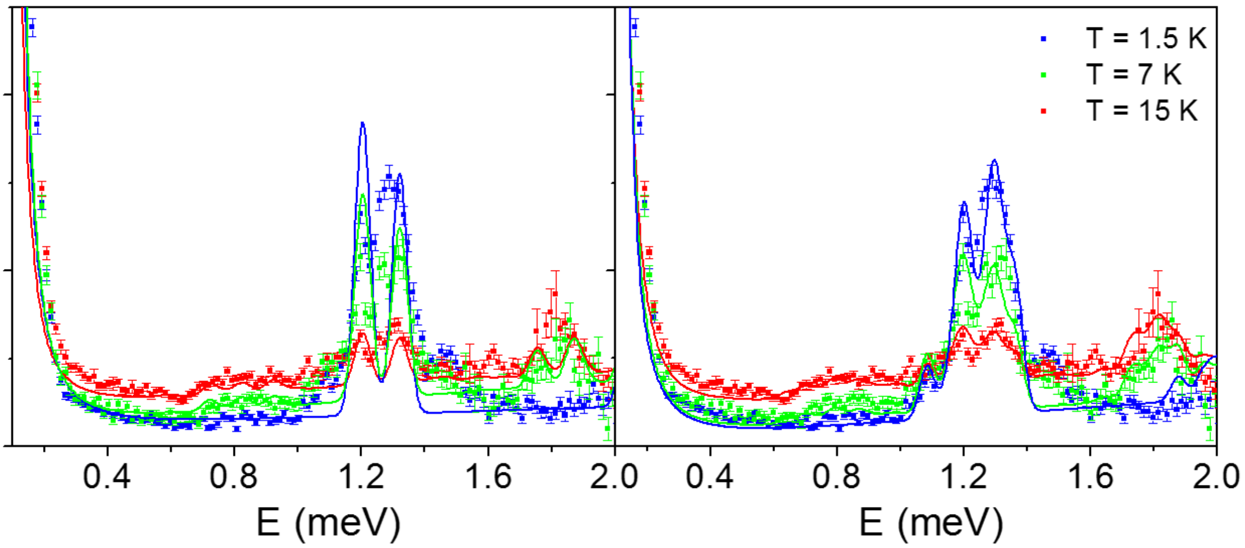

Figure S9: Left: Fitting with an ideally frustrated model (J = 1.35 meV, 10.9 cm^{-1}). Right: fitting with the model reported in the main text (Eq. 1), with J' = 1.2 J, J = 1.32 meV (10.6 cm^{-1}). Measurements performed with 2.5 meV energy of incident neutrons and 58 μeV energy resolution (full width at half maximum at the elastic peak).

In the fit reported in the main text we have assumed easy-axis zero field splitting anisotropy. Indeed, the structural similarity of **1** with other Cr-based rings provides a well-defined starting point. The here-determined value of *d* (-0.022 meV) is in line with those reported for Cr_8 (-0.029 meV)[6] or Cr_8Zn rings (-0.028 meV).[7] However, a good fit can also be obtained with a positive (even if larger) value of *d* (0.048 meV).

In the fitting procedure a Gaussian line shape is associated with each allowed transition, with the full width at half maximum fixed by the value at the elastic peak (apart from the high-resolution E_i = 1.24 meV data, where a larger width is used).

Below we report the dependence of the scattered neutron intensity on the momentum transfer Q for the peak at 1.3 meV (Figure S7), together with the simulation using the model discussed in the main text.

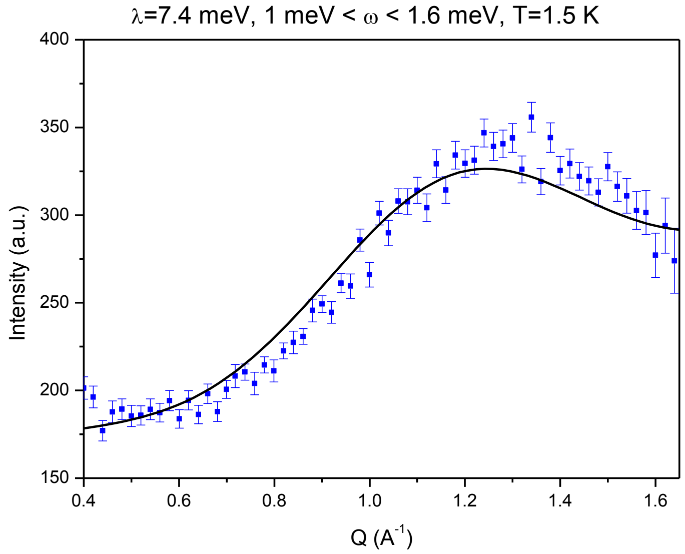

Figure S10: Dependence of the scattered neutron intensity on the momentum transfer Q for the main peak (transition I, S=1/2->S=3/2). There is a good agreement between experimental data (points) and the simulated curve (continuous line), showing that the spatial structure of the states involved in the transition is well reproduced by the model. The curve was calculated using the spin Hamiltonian reported in the text (Eq. 1), with parameters *J*, *J'* and *d* previously obtained from the fit of the energy dependence of the scattered neutron intensity. A contribution quadratic in Q was added to account for the phonon scattering.[8]

5. Classical spin configuration

The classical spin configuration was calculated by treating the spins as classical vectors of length $\sqrt{s(s+1)} = \sqrt{15}/2$ and minimizing the classical (isotropic) energy of the system:

$$E = \sum_{i=1}^{8} J\,\boldsymbol{s}_i \cdot \boldsymbol{s}_{i+1} + J'\,\boldsymbol{s}_9 \cdot \boldsymbol{s}_1$$

Since the interaction is isotropic, there are infinitely many spin configurations minimizing *E* (which depends only on the relative angles between them). The figure reported below was obtained by fixing the position of one of the spins (red arrow). We show two different views of the same spin configuration minimizing *E*. From S11-left, it is clear that all the spins lie on parallel planes, even if the odd number of anti-ferromagnetically coupled spins make the configuration non-collinear (S11-right).

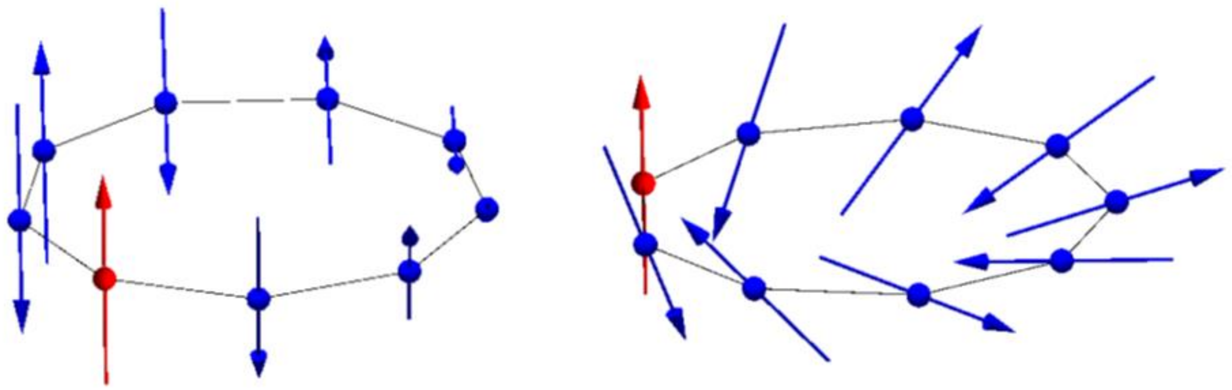

Figure S11. Two views of one of the configurations minimizing the classical energy of the isotropic part of the Hamiltonian, equation (1). The arrows represent Cr spins, with length $\sqrt{s(s+1)} = \sqrt{15}/2$. All the spins belong to the same plane (left), but they are not collinear (right).

5.1. **Spin Chirality**

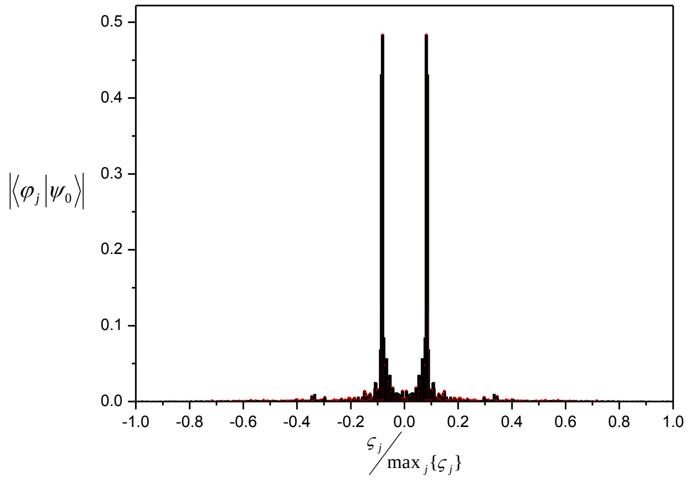

Figure S12. Decomposition of the spin Hamiltonian ground state $|\psi_0\rangle$ onto the eigenstates $|\phi_j\rangle$ of the scalar chirality operator $\hat{C} = \sum_{i=1}^{9} \boldsymbol{s}_i \cdot \boldsymbol{s}_{i+1} \times \boldsymbol{s}_{i+2}$ (i.e., $\hat{C}|\phi_j\rangle = \varsigma_j|\phi_j\rangle$). The quantum ground state is an equal superposition of states with opposite chirality eigenvalues.

6. DFT Calculations

The Density Functional Theory (DFT) calculations reported in this paper were based on the X-ray structure simplified by replacing pivalates with acetates in order to reduce the computational effort, as commonly done for this kind of calculations (see Tomecka *et al.*).[9] We employed NWChem quantum chemistry package[10] and exploit the B3LYP hybrid functional[11] to account for exchange-correlation effects, as typically done in DFT calculations on magnetic molecules.[12] Calculations were performed on the Jülich supercomputer Jureca.

The isotropic exchange couplings were computed by comparing total energy calculations with different spin configurations (according to the broken symmetry approach).[13] Ahlrichs valence triple-ζ (VTZ) basis set for the transition metal ions, and Ahlrichs valence double-ζ (VDZ) basis set for the rest of the elements have been used.[14]

To extract all the nearest-neighbor exchange couplings between Cr ions (s=3/2), we performed total energy calculations on 11 spin configurations: (i) a ferromagnetic arrangement with the maximum projection of the total spin along z (M=27/2); (ii) a staggered state with alternating up and down spins (M=3/2); (iii) nine configurations with eight spins up and one spin down (M=21/2). Energy differences between pairs of these configurations allowed us to extract all the exchange couplings.

SUPPLEMENTARY REFERENCES

1. R.I. Bewley, J.W. Taylor, S.M. Bennington, Nuclear Instruments and Methods in Physics, **2011**, ***637*** 128-134.
2. H. Nowell, S. A. Barnett, K. E. Christensen, S. J. Teat, D. R. Allan, *J. Synchrotron Radiat.* **2012**, *19*, 435-441.
3. (a) L. Krause, R. Herbst-Irmer, G. M. Sheldrick, D. Stalke, *J. Appl. Cryst.* **2015**, *48*, 3-10; (b) R. H. Blessing, *Acta Crystallogr.* **1995**, *A51*, 33-38.
4. (a) G. M. Sheldrick. *Acta Crystallogr.*, **2015,** *C71*, 3-8; (b) O. V. Dolomanov, L. J. Bourhis, R. J. Gildea, J. A. K. Howard, H. Puschmann, *J. Appl. Cryst.* **2009,** *42*, 339–341.
5. PLATON, A Multipurpose Crystallographic Tool (Utrecht University, Utrecht, The Netherlands, 2008).
6. S. Carretta, J. van Slageren, T. Guidi, E. Liviotti, C. Mondelli, D. Rovai, A. Cornia, A. L. Dearden, F. Carsughi, M. Affronte, C. D. Frost, R. E. P. Winpenny, D. Gatteschi, G. Amoretti, and R. Caciuffo, *Phys. Rev. B* **2003**, *67*, 094405.
7. A. Bianchi, S. Carretta, P. Santini, G. Amoretti, T. Guidi, Y. Qiu, J. R. D. Copley, G. Timco, C. Muryn, and R. E. P. Winpenny, *Phys. Rev. B* **2009**, *79*, 144422.
8. S. W. Lovesey, *Theory of Neutron Scattering from Condensed Matter*, Volume 1 (Oxford University Press, New York 1984)
9. D. M. Tomecka, V. Bellini, F. Troiani, F. Manghi, G. Kamieniarz, and M. Affronte, *Phys. Rev. B* **2008**, *77*, 224401.
10. M. Valiev, E.J. Bylaska, N. Govind, K. Kowalski, T.P. Straatsma, H.J.J. van Dam, D. Wang, J. Nieplocha, E. Apra, T.L. Windus, W.A. de Jong, *Comput. Phys. Commun.* **2010**, *181*, 1477.
11. A.D. Becke, *J. Chem. Phys.* **1993**, *98*, 5648.
12. E. Ruiz, S. Alvarez, J. Cano, V. Polo, *J. Chem. Phys.* **2005**, *123*, 164110.
13. L. Noodleman, *J. Chem. Phys.,* **1981**, *74*, 5737.
14. Schafer, A.; Horn, H.; Ahlrichs, R. *J. Chem. Phys.* **1992**, *97*, 2571.
